# Postprandial amino acid, glucose and insulin responses among healthy adults after a single intake of *Lemna minor* in comparison with green peas: a randomised trial

**DOI:** 10.1017/jns.2019.26

**Published:** 2019-08-20

**Authors:** Gertrude G. Zeinstra, Dianne Somhorst, Els Oosterink, Henriette Fick, Ineke Klopping-Ketelaars, Ingrid M. van der Meer, Jurriaan J. Mes

**Affiliations:** 1 Wageningen University and Research, Wageningen Food and Biobased Research, Food, Health & Consumer Research, Bornse Weilanden 9, 6708 WG Wageningen, The Netherlands; 2 Wageningen University and Research, Division of Human Nutrition & Health, Stippeneng 4, 6708 WE Wageningen, The Netherlands; 3 Wageningen University and Research, Wageningen Plant Research, Bioscience, Droevendaalsesteeg 1, 6708 PB Wageningen, The Netherlands

**Keywords:** Duckweed, *Lemna minor*, Plant-based protein, Human trials, Glucose, Insulin, Safety, DIAAS, digestible indispensable amino acid score, EAA, essential amino acids, PDCAAS, protein digestibility-corrected amino acid score, TAA, total amino acids

## Abstract

A high protein content combined with its enormous growth capacity make duckweed an interesting alternative protein source, but information about postprandial responses in humans is lacking. The present study aimed to assess the postprandial serum amino acid profile of *Lemna minor* in healthy adults in comparison with green peas. A secondary objective was to obtain insights regarding human safety. A total of twelve healthy volunteers participated in a randomised, cross-over trial. Subjects received two protein sources in randomised order with a 1-week washout period. After an overnight fast, subjects consumed *L. minor* or peas (equivalent to 20 g of protein). After a baseline sample, blood samples were taken 15, 30, 45, 60, 75, 90, 120, 150 and 180 min after consumption to assess amino acid, glucose and insulin levels. Heart rate, blood pressure and aural temperature were measured before and after consumption, and subjects reported on gastrointestinal discomfort for four subsequent days. Compared with green peas, significantly lower blood concentrations of amino acids from *L. minor* were observed, indicating lower digestibility. *L. minor* consumption resulted in lower plasma glucose and insulin levels compared with peas, probably due to different glucose content. There were no significant differences concerning the assessed health parameters or the number of gastrointestinal complaints, indicating that a single bolus of *L. minor* – grown under controlled conditions – did not induce acute adverse effects in humans. Further studies need to investigate effects of repeated *L. minor* intake and whether proteins purified from *L. minor* can be digested more easily.

In the last decades, the global consumption of animal proteins has increased continuously and is expected to increase further in the coming years. At this moment, animal-derived protein accounts for about 60 % of humans’ total protein consumption in developed countries^(1)^. The increasing standards of living in developing countries and the rapid population growth are the main factors responsible for a higher demand of animal-derived protein^(2–4)^.

The current production of animal-derived protein, even after intensification, would not suffice to keep up with population growth and the associated requirements for amino acids^(4)^. Therefore, a transition towards diets containing more plant-derived protein is needed. This transition will also provide benefits for the environment, animal welfare and human health^(2)^, as a recent study showed that replacing animal protein with plant protein reduced type 2 diabetes risk^(5)^.

To make this shift in diet possible, new plant protein sources have been explored as alternatives, such as (micro)algae, seaweeds, rapeseed and duckweed^(6,7)^. Duckweed has attracted considerable attention for several reasons. First, duckweed has a very high growth rate resulting in a high biomass yield per hectare and can tolerate extreme circumstances^(8–11)^. Second, it can be cultivated outdoors in a basin, in (simple) greenhouses, or in multilayer vertical farming systems, thereby not making use of farming land^(11)^. Third, duckweed contains high amounts of protein (35–43 %) when grown under optimal temperature, light and nutrient availability conditions and harvested regularly^(8,11,12)^. Fourth, duckweed is rich in minerals, vitamins, carotenoids and specific flavonoids and has a favourable fatty acid composition^(12–14)^. Finally, duckweed protein has a better array of essential amino acids (EAA) than most vegetable proteins and more closely resembles animal protein^(10,11)^. Duckweed is also known for its remediation capacity, as metals and other pollutants can be absorbed by the plants. However, this is often in low quantities and foremost caused by growing on contaminated water^(7,12,15)^.

Duckweed has long been used as animal feed. Fresh and dried duckweed has been fed successfully to fish, waterfowl, chickens, cattle, pigs, sheep and goats with good results on growth performance when duckweed was part of their habitual diet^(8,14,16)^, indicating that duckweed can be part of the diet without adverse effects.

Furthermore, duckweed has been eaten by humans for generations as a nutritious vegetable – named ‘Khai-Nam’ – in Southeast Asian countries, including Laos, Thailand and Burma (now Myanmar)^(17)^. In these countries, *Wolffia arrhiza* and *W. globosa* are the dominating species used for human consumption^(12,18)^. So far, no reports of adverse effects of eating duckweed by humans are known^(19)^.

Before implementing duckweed in the human diet, it is important to know the nutritional value in comparison with other plant-based protein sources. However, hardly any information is available on the digestion and nutritional value of proteins from whole vegetables in humans. Most studies on nutritional values have been performed based on isolated protein. The literature has shown that digestibility of these isolated protein in humans can vary between different sources^(20,21)^, which affects amino acid availability in the blood. Digestion values of duckweed protein in animals have been reported to vary between 50 and 88 %^(22,23)^, but no data are available for humans. In addition, different protein sources may also lead to different postprandial glucose and insulin responses^(24,25)^.

Because information about duckweed digestibility in humans is lacking, the primary objective of the present study was to assess the postprandial serum amino acid profile after a single intake of duckweed in healthy adult volunteers in comparison with another high-protein plant source: green peas. Secondary objectives were to assess postprandial plasma glucose and insulin responses and to assess the acute health parameters heart rate, blood pressure and aural temperature and the occurrence of gastrointestinal complaints as part of the safety assessment in humans. The present study was conducted by using *Lemna minor* that showed good growth characteristics when grown indoor in our facilities.

## Methods and materials

### Subjects

Subjects were recruited via the pool of volunteers of Wageningen Food and Biobased Research. Inclusion criteria were: aged between 18 and 50 years, healthy as assessed by a health and lifestyle questionnaire, normal blood pressure, normal blood clinical laboratory tests for Hb, kidney and liver functioning (alanine aminotransferase (ALAT), γ-glutamyl transferase (GGT), creatinine and estimated glomerular filtration rate (eGFR)), appropriate veins for blood sampling and a BMI between 19 and 25 kg/m^2^. Subjects were excluded when they had any metabolic, gastrointestinal, inflammatory or chronic disease (such as diabetes, anaemia, hepatitis, CVD), a history of gastrointestinal surgery, liver dysfunction (cirrhosis, hepatitis), liver surgery or kidney dysfunction (eGFR <60 ml/min). Other exclusion criteria were: use of gastric acid inhibitors or laxatives, use of hard drugs, taking three or more glasses of alcoholic beverages per d, being pregnant or lactating, participating in intense sporting (>16 h per week) and being a current smoker. Subjects who had known allergies towards the products used in the study, or were not willing to consume chicken broth, were also excluded.

**Fig. 1.**
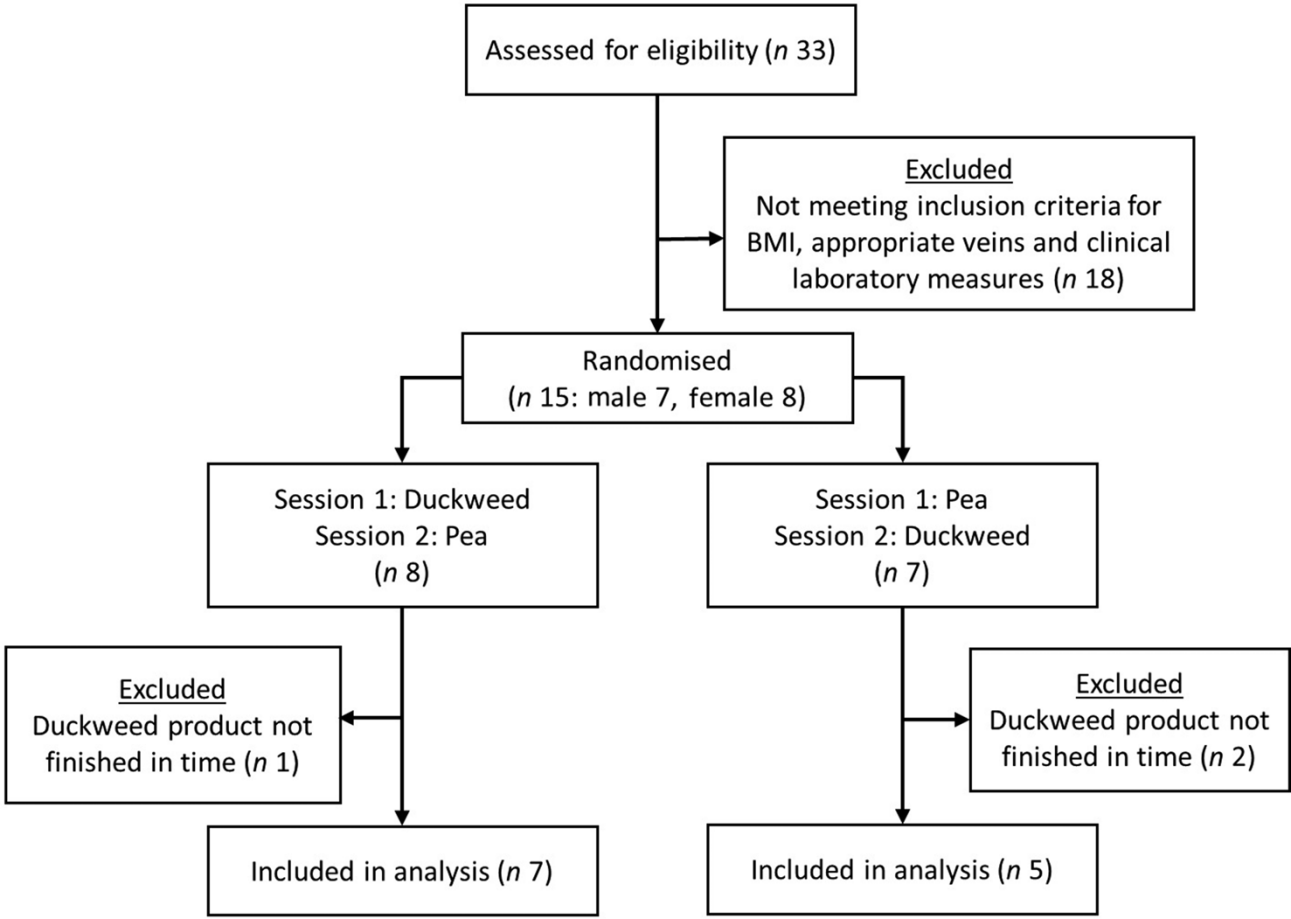
Flow of participants through the randomised trial.

A sample size calculation for paired comparisons with *P* = 0·05 (two-sided, *Z*α = 1·96), *Z*β = 1·28 (power of 90 %), an individual difference in plasma peak values of 100 µg/ml (=sd)^(21,26)^ and a difference of 100 µg/ml regarded as interesting (=δ) indicated that eleven subjects would be sufficient. To account for potential dropout, fifteen subjects were included in the study. Of the participants, three dropped out of the study, because they could not finish the *L*. *minor* product within the given time frame of 20 min. Therefore, the final sample consisted of twelve participants. Fig. 1 shows the flow of the study participants.

### Study design and procedures

The trial was carried out in November 2017. Participants were instructed to avoid alcohol and heavy physical activity for 24 h before the test day, and to maintain their habitual dietary pattern. They received a ready-to-eat pasta meal for the evening before the test day and were instructed to consume this between 18.00 and 20.00 hours (no dessert). Subsequently, participants fasted after this meal (only water or tea allowed without sugar and without milk).

On arrival at the research facilities in Wageningen, The Netherlands, heart rate and blood pressure were measured with an Omron HEM 907 via standardised procedures, as well as aural temperature (to the nearest 0·1 degree). Subsequently, a cannula was inserted for blood sampling. After a baseline blood sample, subjects received the test product warm together with 100 ml water. The *L*. *minor* product had the appearance of a heated portioned frozen spinach, whereas the pea product looked like a bound soup. Subjects were instructed to finish the product within 20 min and spread consumption evenly over this time period. Blood samples were taken at 15, 30, 45, 60, 75, 90, 120, 150 and 180 min after completion. Participants completed a short sensory questionnaire to evaluate the test products. The attributes green colour, intensity of smell, pleasantness of taste, thickness and bitterness of the product were evaluated on a seven-point scale from 1 = not at all to 7 = very much. At the end of the 3 h, heart rate, blood pressure and aural temperature were measured again. In a diary, subjects recorded any gastrointestinal complaints for the test day and the following 3 d. At 1 week later, the same procedures were repeated, but with the other protein source.

The present study was conducted according to the guidelines laid down in the Declaration of Helsinki and all procedures involving human subjects were approved by the Medical Ethical Committee of Wageningen University (protocol no. 17/13). Written informed consent was obtained from all subjects. The study was registered in The Netherlands National Trial Register, reference no. NTR6516 (http://www.trialregister.nl/trialreg/admin/rctview.asp?TC=6516).

### Products

For the present study, *L. minor* (accession 8623) was used. This accession line was obtained in 2011 from a stock culture maintained at the laboratory of Professor Dr Klaus Appenroth (Friedrich Schiller University, Jena), and cultivated and bio-banked at Wageningen Plant Research, Bioscience laboratory from that time on. *L. minor* (8623) plants starting from sterile cultivation were grown in 40-litre containers in an indoor growth chamber on a defined medium (CuSO_4_.5H_2_O, 0·0838 mg/l; H_3_BO, 2·896 mg/l; MnSO_4_.1H_2_O, 2·145 mg/l; Na_2_MoO_4_.2H_2_O, 0·135 mg/l; ZnSO_4_.7H_2_O, 0·536 mg/l; CaCl_2_.2H_2_O, 64·35 mg/l; KH_2_PO_4_, 103·25 mg/l; KNO_3_, 394·25 mg/l; MgSO_4_.7H_2_O, 132·25 mg/l; Fe-EDDHA (ethylenediamine-*N*,*N*′-bis(2-hydroxyphenylacetic acid), 20·45 mg/l). Water was aerated and recycled via UV filters to suppress contaminations in the water. Plants were kept under a day length of 16 h, and day and night temperatures of 23 and 20°C, respectively, a photon flux density of 300–325 µmol/m^2^ per s, a CO_2_ concentration of approximately 400 parts per million and a relative humidity of 75 %. Approximately 40 % of the growth surface were harvested weekly, washed, freeze dried and stored at −20°C. The final batch of *L*. *minor* was analysed in the laboratory for nutritional, microbiological and toxicological content (Nutrilab Giessen; Ansynth Service BV Roosendaal, Wageningen Plant Research and RIKILT-Wageningen). Based on these results, no harm was expected from a single intake in humans. Nutritional content and toxicological measures are shown in the results. The reference product was commercially available green peas (frozen green peas; Bonduelle), which were bought in a local supermarket. Both products were freeze dried and stored in the freezer until use.

The test products were prepared in a research kitchen at the morning of each test day. For an equivalent of 20 g of protein, 64 g of freeze-dried *L*. *minor* or 84 g freeze dried green peas were mixed with water, chicken broth and onion (including totally 4 g butter, 30 g of fresh cut onion, one tablet chicken broth: Maggi; Nestlé) per person portion. The products were cooked for 10 min at 100°C and then served. One portion was 550 g of test product. The test products were coded with a two-digit number and were offered in random order to the participants, on condition that at least two subjects per test day had a similar product. This allocation of treatment to participants was done by a researcher who was not involved in the study. Blinding of the products was not possible, due to an obvious difference in appearance.

### Measurements

The blood samples were stored at −80°C until further analysis. Plasma insulin and glucose levels were assessed in the Gelderse Vallei Hospital in Ede, using standardised clinical procedures. Plasma amino acids were determined at Ansynth Service (Roosendaal) according to the procedure of Terrlink *et al*.^(27)^. Plasma amino acids were analysed by HPLC after precipitating plasma proteins in 50 µl plasma with 200 µl 3 % perchloric acid. Individual amino acids were determined by ultra-fast liquid chromatography (UFLC, an HPLC of Shimadzu) using a pre-column derivatisation with *o*-phthaldialdehyde and fluorimetric detection.

The following gastrointestinal symptoms were included in the participant diary: bloated feeling, belching, flatulence, nausea, abdominal pain, diarrhoea, constipation^(28–31)^. On the test day and the subsequent 3 d, participants scored these symptoms on a seven-point scale with the anchors 1 = not at all present to 7 = strongly present. Adverse events were registered by self-reporting and questioning by a medical doctor. Adverse events were classified under the responsibility of the medical doctor according to 10th revision of the International Statistical Classification of Diseases and Related Health Problems (ICD-10) coding.

### Statistical analysis

Statistical analyses were done using IBM SPSS statistics 23 with a *P* value of *P* < 0·05 considered as statistically significant. Participant characteristics are shown as means with their standard errors (age, BMI) or as percentages (sex).

Blood plasma values that were below the detection threshold were replaced by the half-value of the detection threshold (this was done for the amino acids asparagine acid, hydroxyproline and cysteine, as well as for insulin where <2·0 mU/l was recoded as 1·0). The plasma values of the individual amino acids were summed up to calculate the total amino acids (TAA) and EAA.

For the blood plasma amino acids (TAA, EAA), glucose and insulin, a linear mixed-model analysis for repeated measures with subject as random factor and time (ten levels) and protein source (two levels: *L*. *minor* + peas) as fixed factors was used to assess main effects of time and protein source, as well as an interaction effect time × protein source. In the case of a significant interaction between time and protein source, *post hoc* tests were applied to locate the differences between the two protein sources at each time point with Bonferroni correction (differences were considered significant when *P* < 0·005, in order to correct for multiple comparisons).

In addition, TAA, EAA, glucose and insulin peak values and time to peak were calculated for both protein sources (means and standard errors). A paired *t* test (within-subject design) was used to assess significant differences between the two protein sources in peak value and time to peak.

For aural temperature, blood pressure and heart rate, mean values with their standard errors were calculated per condition (peas *v. L*. *minor*) and per time point (*t* = 0 and *t* = 180). A mixed model with time and protein source as fixed factors and subject as random factor was used to determine main effects of time and protein source as well as the interaction effect time × protein source.

To get insight into the occurrence of gastrointestinal complaints, the scores given by the respondents on the seven-point scale were recoded. A score of five or higher was coded as ‘complaint present’ whereas scores below five were recoded as ‘complaint not present’. Subsequently, the number of gastrointestinal complaints were counted over the 4 d and cross-tabulated per test product. In addition, individual scores per gastrointestinal complaint were averaged across the 4 d per participant. Subsequently, mean scores with their standard errors were calculated per condition and tested with a paired *t* test. Also for the sensory attributes, mean scores with their standard errors were calculated for *L*. *minor* and peas and were compared with a paired *t* test.

## Results

### Macronutrient content

Table 1 shows the general nutrition content, amino acid composition and metal concentrations of *L*. *minor* and green peas in their freeze-dried form. As a comparison, the nutritional composition of egg was added as an example of an animal source. Protein content in *L*. *minor* (31·3 %) is higher than in peas (23·8 %), whereas sugar content is higher in green peas (18·9 %) than in *L*. *minor* (1·2 %). Fibre, starch and fat content are relatively similar in the two products. Egg is fully composed of protein and fat, and only a minor concentration of sugar, and does not contain fibres or starch.

**Table 1.** General nutrition content and metal concentrations of freeze-dried *Lemna minor* and green peas

	*L. minor*	Green peas	Egg*	WHO requirements for adults (g/100 g protein)
Macronutrients (%)				
Protein	31·3	23·8	49·6	
Fat	5·0	3·1	41·7	
Fibre	29·1	24·6	0	
Starch	21·4	14·5	0	
Sugar (glucose)†	1·2	18·9	0·04	
Amino acids (g/100 g protein)				
Threonine (E)	3·5	4·5	4·8	2·3
Tryptophan (E)	1·4	0·6	1·2	0·6
Valine (E)	4·5	5·3	6·1	3·9
Isoleucine (E)	3·6	4·7	5·5	3·0
Leucine (E)	6·6	7·7	8·5	5·9
Phenylalanine (E)	4·4	4·9	5·3	3·0‡
Lysine (E)	4·7	7·5	7·2	4·5
Histidine (E)	1·6	2·0	2·4	1·5
Methionine (E)	1·4	1·0	3·1	1·6
Aspartic acid + asparagine	15·9	10·3	10·0	
Hydroxyproline	<2·00	N.D.	–	
Serine	3·8	4·6	7·4	
Glutamic acid + glutamine	9·1	16·0	13·1	
Proline	3·3	3·8	4·0	
Glycine	4·2	4·1	3·4	
Alanine	5·0	5·6	5·6	
Tyrosine	2·8	3·0	4·1	
Arginine	6·6	10·5	6·0	
Cysteine	1·2	1·1	2·3	
Metals (mg/kg)				
As	0·38	0·040		
Cd§	<2	<1·5		
Hg§	<0·05	<0·05		
Pb§	<3	<2		

E, essential; N.D., non-detectable.

* NDB no. 01129; US Department of Agriculture National Nutrient Database for Standard Reference, Legacy. Current version: April 2018 (http://www.ars.usda.gov/nutrientdata).

† Mono- and disaccharides and reducing sugars conform to the methods NEN 3571 and EU152/2009.

‡ Phenylalanine + tyrosine.

§ Cd, Hg and Pb values were below the detection limit.

The products show a comparable amino acid profile for almost all amino acids and the EAA levels are very close or higher compared with the advised WHO levels^(32)^. The only exception to this is the non-EAA aspartic acid + asparagine, of which the level in *L*. *minor* is relatively high.

In general, metal concentrations in *L*. *minor* were lower than the safe limits of intake, except for As levels which were higher than threshold levels set by the food authorities for rice^(33)^, but of little concern for a single intake.

In some publications, calcium oxalate has been mentioned as a potential risk factor for large-scale and long-term intake in humans, as this component might reduce Ca absorption in the intestine or can induce kidney stones^(34,35)^. The total calcium oxalate content in our batch of *L*. *minor* (209 mg/g freeze-dried duckweed) was much less than in spinach (which is 4·4× higher than *L*. *minor*) and rhubarb (3× higher than *L*. *minor*).

### Subjects

The twelve subjects were on average 31·7 (sem  3·04) years old, had a BMI of 22·7 (sem  0·38) kg/m^2^ and 42 % were female (*n* 5).

### Plasma amino acid profile after consumption of the two protein products

**Fig. 2.**
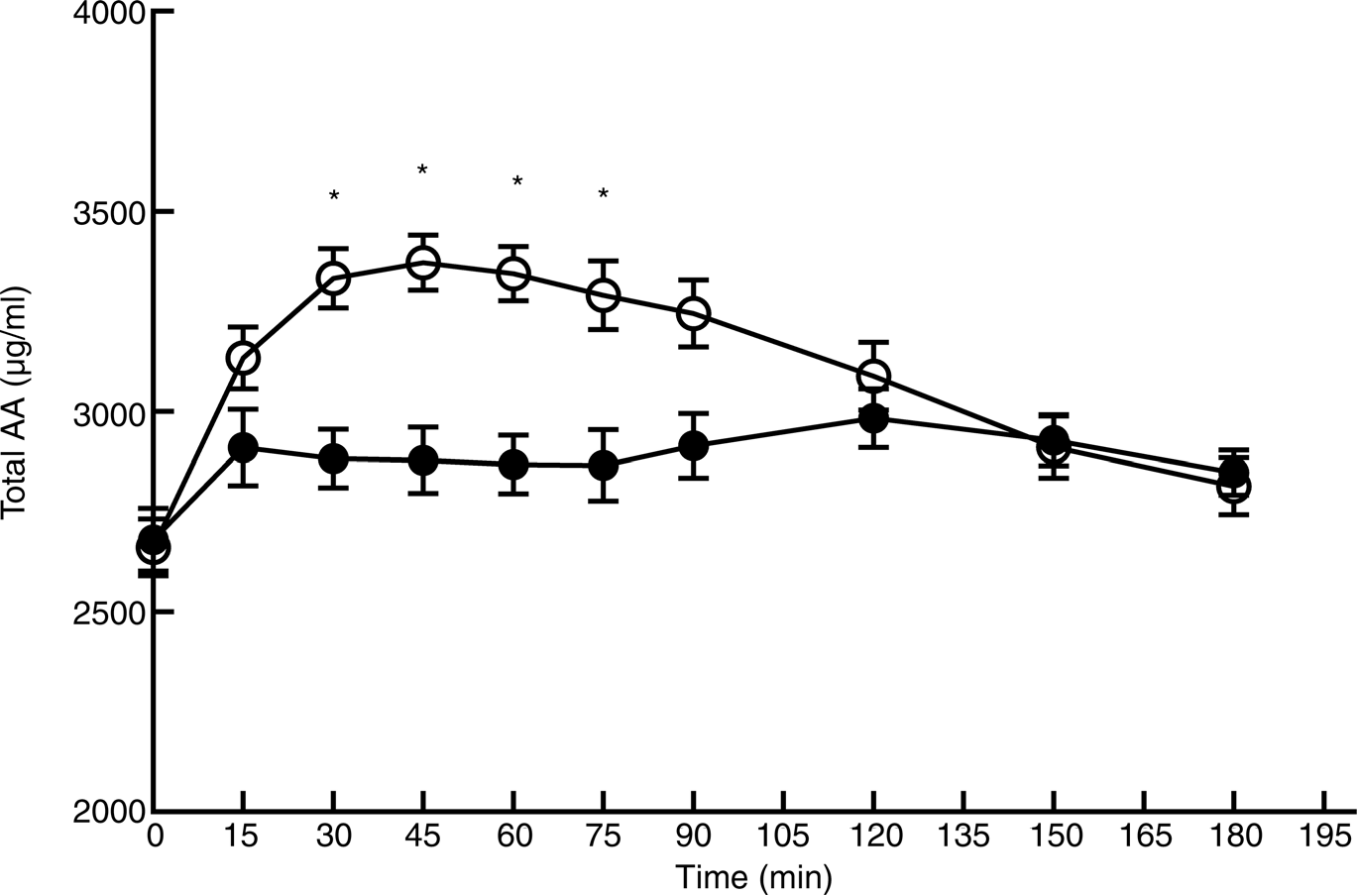
Plasma amino acid profile for total amino acids (AA) after consumption of *Lemna minor* (–●–) and green peas (–○–). Values are means, with standard errors represented by vertical bars. * Significant difference at *P* < 0·005 (*post hoc t* test).

**Fig. 3.**
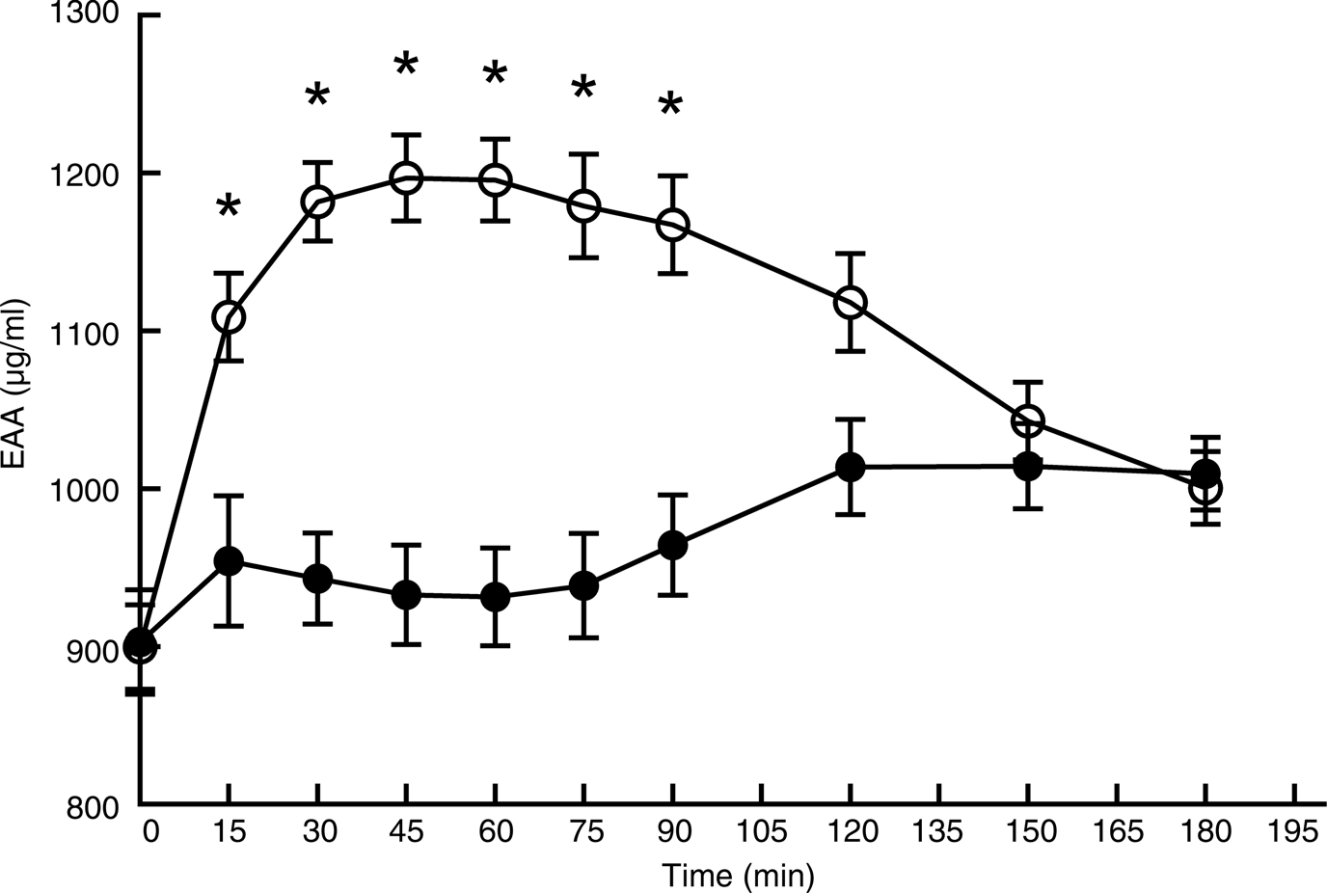
Plasma amino acid profile for essential amino acids (EAA) after consumption of *Lemna minor* (–●–) and green peas (–○–). Values are means, with standard errors represented by vertical bars. * Significant difference at *P* < 0·005 (*post hoc t* test).

Figs 2 and 3 show the profile for serum concentrations of TAA and EAA after consumption of *L*. *minor* or peas. Baseline values (T0) were not significantly different between *L*. *minor* and peas for TAA (*P* = 0·82) and EAA (*P* = 0·88). For peas, TAA rose sharply after ingestion and decreased again after 45 min, whereas for *L*. *minor*, TAA increased only slightly and remained relatively stable. The figures obviously show that the AUC is lower for *L*. *minor* compared with green peas, indicating that *L*. *minor* protein is not well digested and absorbed.

The mixed-model analysis for TAA showed a significant main effect of time (*F*_(9,206)_ = 14·76; *P* < 0·0001), treatment (*F*_(1,206)_ = 102·70; *P* < 0·0001) and interaction (*F*_(9,206)_ = 8·18; *P* < 0·0001). Pairwise comparisons showed significant differences between *L*. *minor* and peas at four time points. A similar picture was seen for EAA, with a significant main effect of time (*F*_(9,206)_ = 14·87; *P* < 0·0001), treatment (*F*_(1,206)_ = 309·44; *P* < 0·0001) and interaction (*F*_(9,206)_ = 17·28; *P* < 0·0001). EAA levels for *L*. *minor* and peas were significantly different at six time points (see Fig. 3).

Peak values and time to peak for TAA and EAA are shown in Table 2. Peak values for EAA and TAA were significantly higher for peas compared with *L*. *minor*, whereas time to peak was approximately twice as high for *L*. *minor* for both EAA and TAA. These results confirm the figures, indicating that *L*. *minor* protein appears in the blood later and with a lower peak.

**Table 2.** Peak values and time to peak for total amino acids (TAA), essential amino acids (EAA), glucose and insulin after ingestion of *Lemna minor* and green peas (Mean values with their standard errors; *n* 12)

	*L. minor*		Green peas		
	Mean	sem	Mean	sem	P*
TAA					
Peak value TAA (μg/ml)	3014·64	66·99	3429·32	74·56	0·001
TAA time to peak (min)	96·25	14·94	46·25	5·68	0·02
EAA					
Peak value EAA (μg/ml)	1033·43	28·14	1224·31	26·99	<0·001
EAA time to peak (min)	150·00	7·39	62·50	7·80	<0·001
Glucose					
Peak value glucose (mmol/l)	5·29	0·19	5·44	0·13	0·56
Glucose time to peak (min)	33·75	12·68	35·00	16·03	0·95
Insulin					
Peak value insulin (mU/l)	12·06	1·75	27·61	2·20	<0·001
Insulin time to peak (min)	33·75	6·16	16·25	1·25	0·023

* By *t* test.

For both *L*. *minor* and green peas, the first limiting amino acid based on the peak value of the postprandial amino acid levels was calculated to be methionine. This was especially true for *L*. *minor*, where methionine was 15× lower than the WHO recommendation, whereas for peas, methionine was five times lower.

### Plasma glucose and insulin profile after ingestion of the two protein products

**Fig. 4.**
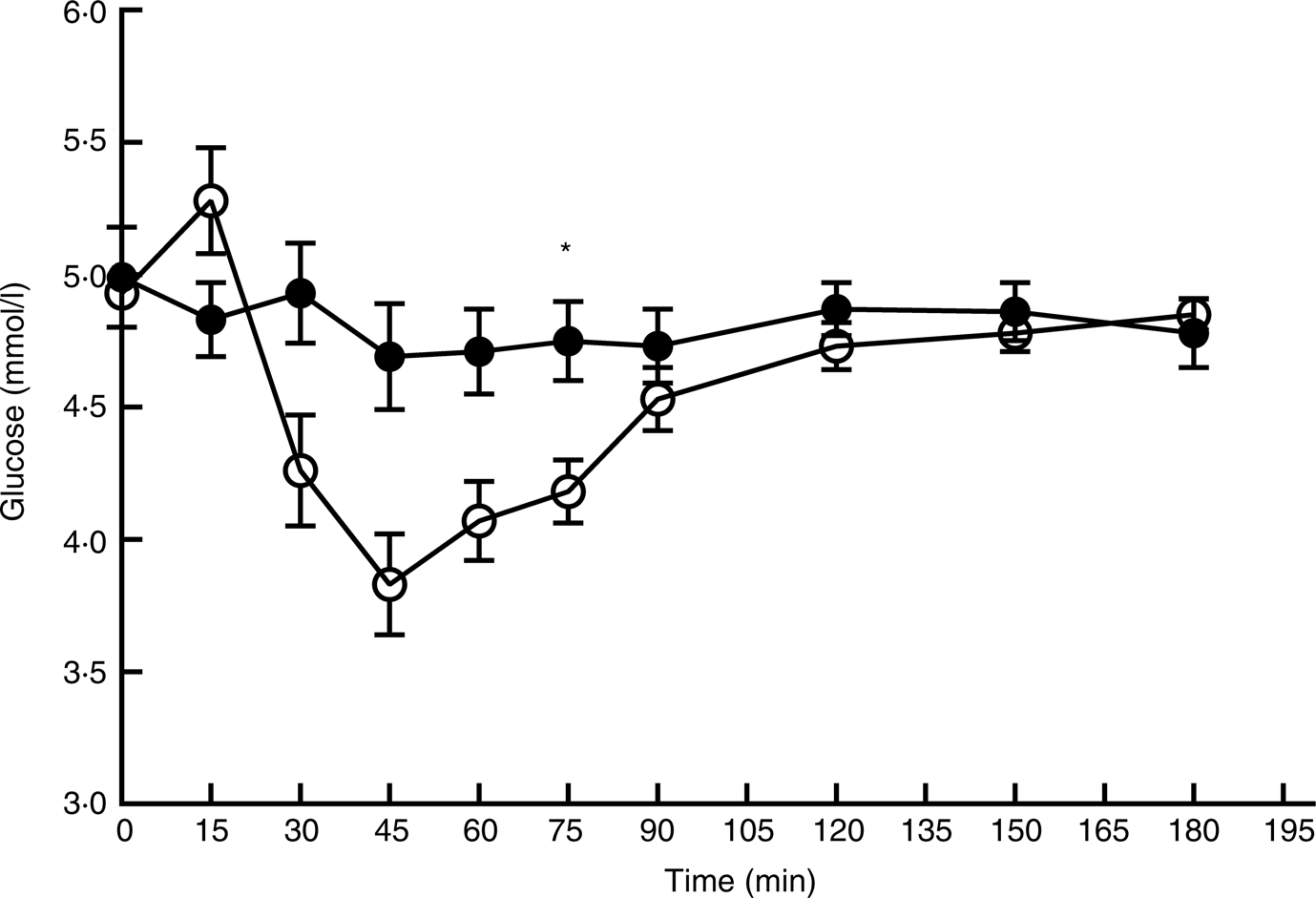
Plasma glucose profile after consumption of *Lemna minor* (–●–) and green peas (–○–). Values are means, with standard errors represented by vertical bars. * Significant difference at *P* < 0·005 (*post hoc t* test).

**Fig. 5.**
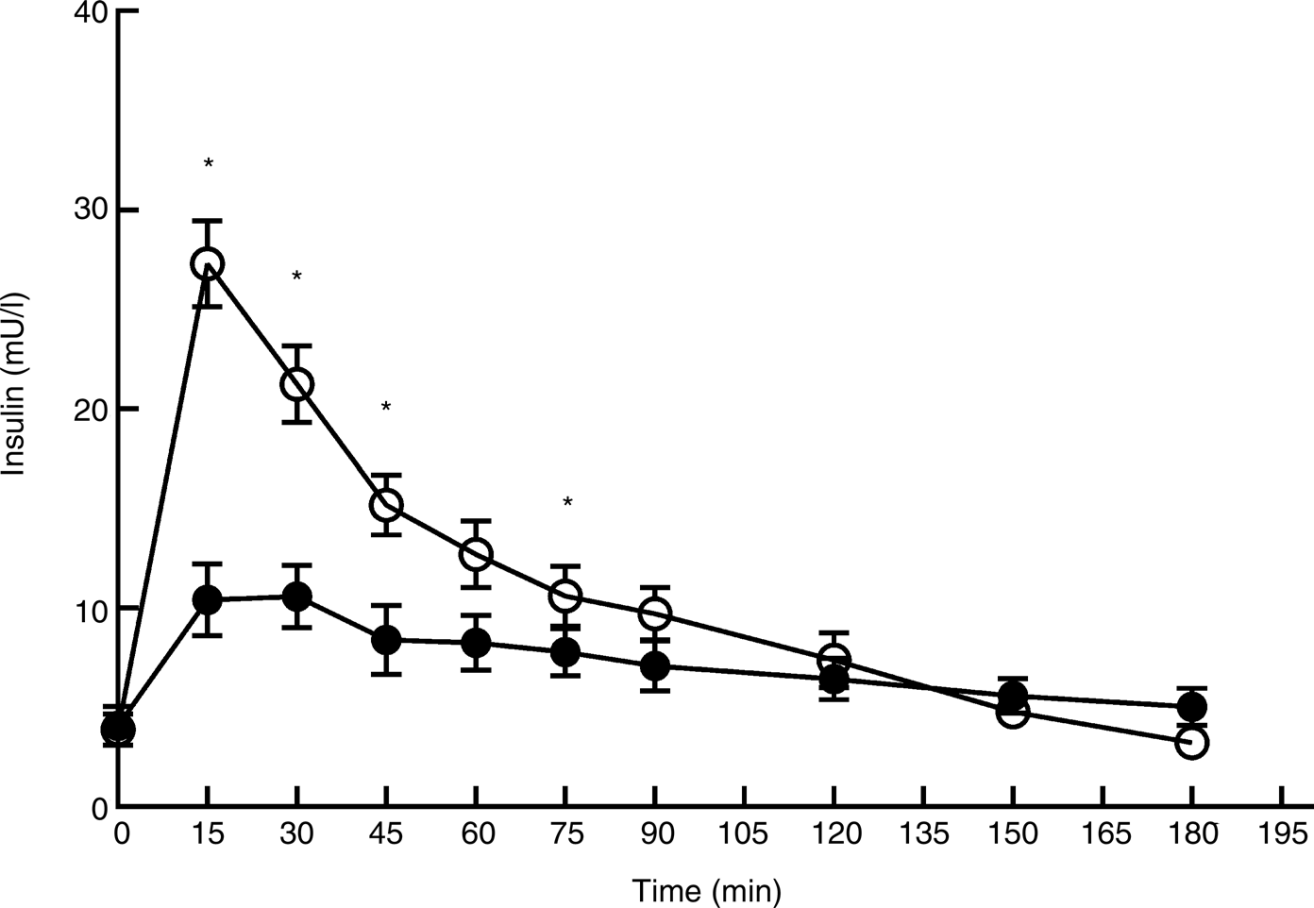
Plasma insulin profile after consumption of *Lemna minor* (–●–) and green peas (–○–). Values are means, with standard errors represented by vertical bars. * Significant difference at *P* < 0·005 (*post hoc t* test).

The baseline values (T0) for glucose and insulin were not significantly different between *L*. *minor* and peas (*P* = 0·75 and *P* = 0·74, respectively). For glucose (Fig. 4), there was a significant main effect of time (*F*_(9,206)_ = 10·03; *P* < 0·0001), treatment (*F*_(1,206)_ = 28·12; *P* < 0·0001) and interaction (*F*_(9,206)_ = 5·56; *P* < 0·0001). Whereas glucose levels remained relatively stable after *L*. *minor* consumption, glucose peaks at *t* = 15 after consumption of peas and then drops quickly until *t* = 45, after which glucose levels stabilise again. Also for insulin (Fig. 5), there was a significant main effect of time (*F*_(9,206)_ = 68·52; *P* < 0·0001), treatment (*F*_(1,206)_ = 124·23; *P* < 0·0001) and interaction (*F*_(9,206)_ = 21·53; *P* < 0·0001). Again, insulin levels remain relatively stable after ingestion of *L*. *minor*, but sharply peak after ingestion of green peas. These results indicate that the postprandial glucose and insulin responses are different after ingestion of *L*. *minor* and pea products.

There were no significant differences between *L*. *minor* and peas for the peak values of blood glucose and the time to peak for glucose (see Table 2). Peak values of insulin were higher for peas (*P* < 0·001) compared with *L*. *minor* and time to peak for insulin was higher for *L*. *minor* (*P* = 0·02). So, after ingestion of *L*. *minor*, insulin levels peak lower and later than after ingestion of peas.

### Blood pressure, heart rate and temperature

Table 3 shows the mean values for blood pressure, heart rate and aural temperature before and after consumption of the two products. A mixed model with time (T0 and T180) and protein type (peas and *L*. *minor*) as fixed factors and subject as random factor showed no significant interaction effect (*F*_(1,33)_ = 0·02; *P* = 0·90). The main effects were also non-significant (time: *F*_(1,33)_ = 0·21, *P* = 0·65; protein type: *F*_(1,34)_ = 1·22, *P* = 0·28). So, there were no significant differences in these health parameters between the two time points or between the two protein-rich products.

**Table 3.** Blood pressure, heart rate and aural temperature before (T0) and at 3 h (T180) after consumption of the two protein products (Mean values with their standard errors; *n* 12)

	*Lemna minor*				Green peas			
	T0		T180		T0		T180	
	Mean	sem	Mean	sem	Mean	sem	Mean	sem
Systolic blood pressure (mmHg)	122·83	3·20	121·50	4·34	120·00	3·67	119·25	2·92
Diastolic blood pressure (mmHg)	64·42	2·43	65·17	2·94	60·25	2·24	62·17	2·25
Heart rate (bpm)	62·08	2·46	58·00	1·85	60·50	2·28	57·83	3·04
Aural temperature (°C)	36·20	0·06	36·66	1·69	36·26	0·09	36·66	0·09

bpm, Beats per min.

### Gastrointestinal symptoms

No serious adverse events were observed during the study. The occurrence of gastrointestinal symptoms over the 4 d after consumption was relatively low, with no clinically relevant differences in occurrence between the two products.

After consuming the peas, six complaints were reported by two subjects (5 × flatulence and 1 × diarrhoea). After consuming *L*. *minor*, ten complaints were reported by four subjects (2 × belching, 1 × flatulence, 2 × nausea, 4 × abdominal pain and 1 × diarrhoea).

Table 4 confirms the low incidence of gastrointestinal symptoms. For both products, average scores per symptom were all below a score of two on a seven-point scale, with no significant differences between *L*. *minor* and peas (all *P* > 0·12).

**Table 4.** Average scores* for the gastrointestinal symptoms that were reported by the participants in the 4 d after consuming the two protein products (Mean values with their standard errors; *n* 12)

	*Lemna minor*		Green peas		
	Mean	sem	Mean	sem	*P*†
Bloated feeling	1·46	0·13	1·56	0·23	0·64
Belching	1·46	0·14	1·25	0·13	0·12
Flatulence	1·81	0·30	2·04	0·44	0·56
Nausea	1·56	0·24	1·17	0·11	0·18
Abdominal pain	1·60	0·22	1·25	0·10	0·15
Diarrhoea	1·40	0·18	1·42	0·22	0·91
Constipation	1·23	0·14	1·23	0·13	1·0

* 1 = Not at all present; 7 = strongly present.

† By *t* test.

### Sensory evaluation

Table 5 shows the mean scores for the sensory attributes of the two protein products. *L*. *minor* was significantly less liked (*P* < 0·001) and evaluated as more bitter (*P* = 0·003) compared with the green pea product. Surprisingly, despite the apparent difference in texture, thickness was not perceived as significantly different.

**Table 5.** Sensory characteristics of the *Lemna minor* and green pea products (Mean values with their standard errors; *n* 12)

	*L. minor*		Green peas		
	Mean	sem	Mean	sem	P*
Green colour	7·92	0·43	8·17	0·27	0·60
Intensity of smell	5·00	0·70	4·50	0·53	0·59
Pleasantness of taste	3·33	0·53	7·33	0·28	<0·001
Thickness	8·25	0·51	7·17	0·34	0·16
Bitterness	3·83	0·59	1·92	0·23	0·003

* By *t* test.

## Discussion

The primary objective of the present study was to analyse the protein uptake from *L*. *minor* plant material as human food by the means of postprandial serum amino acid profile after a single intake in healthy adult volunteers in comparison with green peas. To our knowledge, this is the first study that evaluated the postprandial effects of *L*. *minor* consumption in humans.

The protein content in our batch of plant material (31·3 %) falls within the range that previous papers stated for *Lemna* (25–43 %)^(8,11,12)^. Fat content was comparable to the *L*. *minor* results of Appenroth *et al.*^(12)^, whereas starch content was much higher in our batch of *L*. *minor* (21·4 *v.* 4 %) which can be caused by differences in environmental conditions, growth medium and/or accession used. The amino acid composition of our product (analysed as full product, not as protein extract) showed some small differences compared with the literature; however, when comparing the EAA, our data give a 0·96 correlation with values for *L*. *minor* of both Dewanji^(36)^ and Appenroth *et al.*^(12)^. Comparison of the amino acid composition with the amino acid requirements for adults^(32)^ indicates that the concentrations of these EAA in our batch were all slightly higher than the requirements for adults except for methionine (1·6 g/100 g protein WHO *v.* 1·4 g/100 g protein in *L*. *minor*). Overall, it can be concluded that *L*. *minor* is an interesting protein source from a nutritional content perspective.

The present study showed large differences between duckweed and peas in the postprandial plasma levels of amino acids. This implies that digestion and absorption of protein from this large bolus of *L*. *minor* plant material is relatively low compared with peas. This low digestion may be explained by anti-nutritional factors in the *L*. *minor*, such as trypsin inhibitor, calcium oxalate, tannin and phytate^(37)^. Also animal studies indicate that anti-nutritional factors may be present which can limit intake and growth when animals are fed duckweed at high levels^(8,38)^.

Hardly any publications are available regarding the digestion and uptake of protein from whole food products like vegetables, especially leafy vegetables, based on similar human studies. Most studies assessing postprandial amino acids profiles are based on isolated protein extract which will be quite different from whole products as firmness of cell walls, presence of dietary fibres, anti-nutritional factors, freeze drying and other process or matrix effects can have a large effect on the digestibility and bioavailability of nutrients^(39,40)^. This is nicely illustrated by the study of Rutherfurd *et al.*^(41)^ in which the protein quality of peas and isolated protein from the same peas were analysed. In the present study, both the PDCAAS (protein digestibility-corrected amino acid scores) and DIAAS (digestible indispensable amino acid scores) as prescribed by the FAO^(42)^ were used to quantify the protein quality. The assessed PDCAAS for cooked peas was 0·597 and the DIAAS 0·579, being much lower than what was found for pea protein concentrate for which a PDCAAS of 0·893 and DIAAS of 0·822 were found, indicating a clear effect of the matrix on digestibility.

The observed differences in amino acid availability for the two plant sources *L*. *minor* and peas are only relevant for populations with a marginal protein intake, for whom the consequences of overestimating protein quality may be severe. In general, the Western population eats more protein than is minimally recommended^(43,44)^ and no direct problems can be expected when not being able to release all proteins from a food product consumed. In this respect, it should be noted that the present study does not give insight in the digestion rate of a normal portion size. The 20 g duckweed protein consumed here in one bolus equals 1111 g of fresh *L*. *minor* material, a portion size not normally consumed in one meal. Some of the study participants had a hard time finishing the large portion in time. Three participants could not finish the duckweed portion in time and were omitted from the data analysis. Potentially, the firmness of the product and the intense chewing that was required caused some sensory satiation^(45)^ together with a full stomach, resulting in a reluctance to eat further and being unable to finish the portion in time. As bulk size may influence the efficiency of digestion^(46)^, it would be interesting to study whether normal portion sizes of *L*. *minor* as vegetable have a relatively better digestion and uptake than represented here in the present study.

The different postprandial glucose and insulin responses after ingestion of *L*. *minor* and peas in the present study can be explained by the different sugar levels in the raw product. The sugar content of freeze-dried green peas was 18·9 %, whereas the sugar content of *L*. *minor* was only 1·2 %. The used portion per person therefore equals 15·9 g sugars for peas and 0·8 g for *L*. *minor*. This large difference obviously explains the differences in postprandial glucose and insulin responses, and implies that consumption of *L*. *minor* will hardly have any impact on blood insulin levels.

### Limitations and strengths

A strength of the present study is that we demonstrated differences in protein digestibility of whole plant products in humans by a straightforward human trial design. Although not as exact as studies based on labelled proteins, the present study indicates clear differences in digestion and uptake when offering a bolus that equals 20 g of protein present in different products. In the present study, we compared the bioavailability of plant protein present in a seed crop (peas) with proteins present in a ‘leaf crop’ (*L*. *minor*). The lower bioavailability of *Lemna* protein compared with peas warrants further research on the digestibility of other leafy vegetables by the same study design. We argue that such studies should be performed systematically for food products (and not only protein isolates) and especially new protein-rich food products, such as those that replace animal-based proteins, in order to gain insight into the true nutritional impact of these products. The measurements of amino acids, glucose, insulin, the various health parameters and gastrointestinal complaints add to obtaining a comprehensive picture, which shows that one very large intake of *Lemna* plants – grown under controlled conditions – may not lead to high postprandial amino acids levels and was well tolerated by healthy adults.

A limitation of the present study is the fact that blinding of the treatment was not possible because whole food products were tested which are easy recognised by shape, taste and texture. The *L*. *minor* and pea products could not be identically matched on sensory attributes, so the participants could recognise both products. Despite an obvious difference between the two products regarding texture, this texture difference was not reported by the respondents in the sensory evaluation. Yet, they reported a lower liking for the *L*. *minor* product and a higher score for bitterness. Recognition of the product could have affected the number of gastrointestinal complaints reported as subjects might have been more on guard when consuming an uncommon food product, such as *L*. *minor*. However, no significant differences were found between the two protein sources, so it seems likely that these sensory differences did not influence our gastrointestinal results. A second limitation is that we have no information about the amino acid profiles after 180 min and we therefore recommend expanding sampling time when analysing protein products with an expected low digestion and uptake rate.

### Implications and recommendations

The proteins in the *L*. *minor* plants seem to be less easily available, resulting in a lower digestibility, and a subsequent lower appearance in the blood. This product will be of interest for the protein transition to increase the sustainability of consumer diets, especially in developed countries, where efficient utilisation of proteins due to underweight or protein malnutrition is less of an issue. Because consumers generally respond positively towards trying and consuming duckweed^(47)^, duckweed might have great potential to become a widely accepted food source. However, due to the low protein digestibility of *L*. *minor* as a plant, it is recommended to also investigate the digestibility of isolated *Lemna* protein, because this is probably easier to digest and absorb as shown for other protein extracts and hydrolysates^(40)^. However, before *Lemna* plants or proteins can be part of the habitual human diet, a successful Novel Food Application should be completed.

The present study indicates that a single, relatively large bolus of *L*. *minor* plants is well tolerated by humans when it is grown under well-controlled circumstances. Nevertheless, *Lemna* can accumulate heavy metals even when grown on high-quality tap water as available in The Netherlands. The amounts detected in the plant in our batch were not expected to be threatening when taken at a single occasion. Still, in subsequent studies, demi-water or source water and fertilisers with low concentrations of undesired metals and certain minerals should be used to minimise accumulation in the plants. Regularly measuring metal concentrations in the water and harvested plants should be part of the standard quality and safety analysis for *L*. *minor*. In earlier studies, calcium oxalate content of duckweed grown under natural conditions in the wild was reported as potentially harmful. Oxalate may hinder the Ca uptake in the body and may increase the risk of kidney stones^(34,35)^. The batch in the present study contained less oxalate than in commercially available spinach and rhubarb, and provided no risk from a single intake. Subsequent studies should investigate the digestion and safety effects of repeated consumption of *L*. *minor*.

### Conclusion

The results of this human trial showed that proteins from *L*. *minor* consumed as whole plant product end up in the blood at lower concentrations compared with green peas, indicating low protein digestibility in humans. Due to the lower sugar content, *L*. *minor* had more positive plasma profiles for glucose and insulin compared with peas. There were no significant differences between the two protein-rich products regarding the assessed health parameters or the number of gastrointestinal complaints, providing the first indications that consuming a single meal of *L*. *minor* – grown under strict controlled circumstances – is well tolerated by humans.

## References

[1] Pasiakos SM, Agarwal S, Lieberman HR, (2015) Sources and amounts of animal, dairy, and plant protein intake of US adults in 2007–2010. Nutrients 7, 7058–7069. 26308049 10.3390/nu7085322 PMC4555161

[2] Aiking H (2011) Future protein supply. Trends Food Sci Technol 22, 112–120.

[3] Boland MJ, Rae AN, Vereijken JM, (2013) The future supply of animal-derived protein for human consumption. Trends Food Sci Technol 29, 62–73.

[4] Gilland B (2002) World population and food supply: can food production keep pace with population growth in the next half-century? Food Policy 27, 47–63.

[5] Virtanen HEK, Koskinen TT, Voutilainen S, (2017) Intake of different dietary proteins and risk of type 2 diabetes in men: the Kuopio Ischaemic Heart Disease Risk Factor Study. Br J Nutr 117, 882–893. 28397639 10.1017/S0007114517000745

[6] Van der Peet G & Kamp J (2011) Workshop Nieuwe Kansen Voor Eiwit (New Opportunities for Protein Workshop). Wageningen: Wageningen UR Livestock Research.

[7] van der Spiegel M, Noordam MY & van der Fels-Klerx HJ (2013) Safety of novel protein sources (insects, microalgae, seaweed, duckweed, and rapeseed) and legislative aspects for their application in food and feed production. Compr Rev Food Sci Food Saf 12, 662–678. 10.1111/1541-4337.12032 33412718

[8] Goopy JP & Murray PJ (2003) A review on the role of duckweed in nutrient reclamation and as a source of animal feed. Asian-Australas J Anim Sci 16, 297–305.

[9] Hassan MS & Edwards P (1992) Evaluation of duckweed (*Lemna perpusilla* and *Spirodela polyrrhiza*) as feed for Nile tilapia (*Oreochromis niloticus*). Aquaculture 104, 315–326.

[10] Iqbal S (1999) Duckweed Aquaculture. Potentials: Possibilities and Limitations for Combined Wastewater Treatment and Animal Feed Production in Developing Countries, SANDEC report no. 6/99 Duebendorf, Switzerland: Department of Water & Sanitation in Developing Countries (SANDEC) and Swiss Federal Institute for Environmental Science & Technology (EAWAG).

[11] Leng RA, Stambolie JH & Bell R (1995) Duckweed – a potential high-protein feed resource for domestic animals and fish. Livest Res Rural Dev 7, 5.

[12] Appenroth K-J, Sree KS, Böhm V, (2017) Nutritional value of duckweeds (Lemnaceae) as human food. Food Chem 217, 266–273. 27664634 10.1016/j.foodchem.2016.08.116

[13] Edelman M & Colt M (2016) Nutrient value of leaf vs. seed. Front Chem 4, 32–32. 27493937 10.3389/fchem.2016.00032 PMC4954856

[14] Mwale M & Gwaze FR (2013) Characteristics of duckweed and its potential as feed source for chickens reared for meat production: a review. Sci Res Essays 8, 689–697.

[15] Leng RA (1999) Duckweed: A Tiny Aquatic Plant with Enormous Potential for Agriculture and Environment. Rome: FAO.

[16] Tavares FDA, Rodrigues JBR, Fracalossi DM, (2008) Dried duckweed and commercial feed promote adequate growth performance of tilapia fingerlings. Biotemas 21, 91–97.

[17] Bhanthumnavin K & McGarry MG (1971) *Wolffia arrhiza* as a possible source of inexpensive protein. Nature 232, 495. 4937216 10.1038/232495a0

[18] Sree KS & Appenroth KJ (2016) Duckweed science and food excursion in Thailand. In *Duckweed Forum*, vol. 4, pp. 274–275. International Steering Committee on Duckweed Research and Applications http://www.ruduckweed.org/uploads/1/0/8/9/10896289/iscdra-duckweedforum_issue14-2016-07-corrected.pdf?

[19] Appenroth KJ & Sree KS (2016) Duckweed for human nutrition In Duckweed Forum, vol. 4, pp. 313–314. International Steering Committee on Duckweed Research and Applications http://www.ruduckweed.org/uploads/1/0/8/9/10896289/iscdra-duckweedforum_issue15-2016-010.pdf

[20] Bos C, Metges CC, Gaudichon C, (2003) Postprandial kinetics of dietary amino acids are the main determinant of their metabolism after soy or milk protein ingestion in humans. J Nutr 133, 1308–1315. 12730415 10.1093/jn/133.5.1308

[21] Farnfield MM, Trenerry C, Carey KA, (2009) Plasma amino acid response after ingestion of different whey protein fractions. Int J Food Sci Nutr 60, 476–486. 18608553 10.1080/09637480701833465

[22] Damry, Nolan JV, Bell RE, (2001) Duckweed as a protein source for fine-wool merino sheep: its edibility and effects on wool yield and characteristics. Asian-Australas J Anim Sci 14, 507–514.

[23] Du Thanh H, Nguyen Quang L, Everts H, (2009) Ileal and total tract digestibility in growing pigs fed cassava root meal and rice bran with inclusion of cassava leaves, sweet potato vine, duckweed and stylosanthes foliage. Livest Res Rural Dev 21, 12.

[24] Comerford K & Pasin G (2016) Emerging evidence for the importance of dietary protein source on glucoregulatory markers and type 2 diabetes: different effects of dairy, meat, fish, egg, and plant protein foods. Nutrients 8, E446. 27455320 10.3390/nu8080446 PMC4997361

[25] Crowder CM, Neumann BL & Baum JI (2016) Breakfast protein source does not influence postprandial appetite response and food intake in normal weight and overweight young women. J Nutr Metab 2016, 6265789. 26885386 10.1155/2016/6265789 PMC4739264

[26] He T, Spelbrink RE, Witteman BJ, (2013) Digestion kinetics of potato protein isolates *in vitro* and *in vivo*. Int J Food Sci Nutr 64, 787–793. 23713493 10.3109/09637486.2013.793300

[27] Terrlink T, van Leeuwen PA & Houdijk A (1994) Plasma amino acids determined by liquid chromatography within 17 min. Clin Chem 40, 245–249. 8313601

[28] Bovenschen HJ, Janssen MJ, van Oijen MG, (2006) Evaluation of a gastrointestinal symptoms questionnaire. Dig Dis Sci 51, 1509–1515. 16927133 10.1007/s10620-006-9120-6

[29] Luiking YC, Abrahamse E, Ludwig T, (2016) Protein type and caloric density of protein supplements modulate postprandial amino acid profile through changes in gastrointestinal behaviour: a randomized trial. Clin Nutr 35, 48–58. 25790724 10.1016/j.clnu.2015.02.013

[30] van Loon LJ, Saris WH, Verhagen H, (2000) Plasma insulin responses after ingestion of different amino acid or protein mixtures with carbohydrate. Am J Clin Nutr 72, 96–105. 10871567 10.1093/ajcn/72.1.96

[31] Veenstra JM, Duncan AM, Cryne CN, (2010) Effect of pulse consumption on perceived flatulence and gastrointestinal function in healthy males. Food Res Int 43, 553–559.

[32] Food and Agriculture Organization of the United Nations, World Health Organization & United Nations University (2007) Protein and Amino Acid Requirements in Human Nutrition: Report of a Joint FAO/WHO/UNU Expert Consultation. WHO Technical Report Series 935. Geneva: WHO.

[33] European Commission (2015) Verordening (EU) 2015/1006 van de Commissie van 25 juni 2015 tot wijziging van Verordening (EG) nr. 1881/2006 wat de maximumgehalten voor anorganisch arseen in levensmiddelen betreft (Regulation for maximum allowances of arsenic in food products) https://publications.europa.eu/en/publication-detail/-/publication/4ea62ae9-1bc8-11e5-a342-01aa75ed71a1/language-nl

[34] Champ MMJ (2002) Non-nutrient bioactive substances of pulses. Br J Nutr 88, S307–S319. 12498631 10.1079/BJN2002721

[35] Noonan SC & Savage GP (1999) Oxalate content of foods and its effect on humans. Asia Pac J Clin Nutr 8, 64–74. 24393738

[36] Dewanji A (1993) Amino acid composition of leaf proteins extracted from some aquatic weeds. J Agric Food Chem 41, 1232–1236.

[37] Kalita P, Mukhopadhyay PK & Mukherjee AK (2007) Evaluation of the nutritional quality of four unexplored aquatic weeds from northeast India for the formulation of cost-effective fish feeds. Food Chem 103, 204–209.

[38] Solomon SG & Okomoda VT (2012) Growth performance of *Oreochromis niloticus* fed duckweed (*Lemna minor*) based diets in outdoor hapas. Int J Res Fish Aquacult 2, 61–65.

[39] Millward DJ, Layman DK, Tomé D, (2008) Protein quality assessment: impact of expanding understanding of protein and amino acid needs for optimal health. Am J Clin Nutr 87, 1576S–1581S. 18469291 10.1093/ajcn/87.5.1576S

[40] Morifuji M, Ishizaka M, Baba S, (2010) Comparison of different sources and degrees of hydrolysis of dietary protein: effect on plasma amino acids, dipeptides, and insulin responses in human subjects. J Agric Food Chem 58, 8788–8797. 20614926 10.1021/jf101912n

[41] Rutherfurd SM, Fanning AC, Miller BJ, (2015) Protein digestibility-corrected amino acid scores and digestible indispensable amino acid scores differentially describe protein quality in growing male rats. J Nutr 145, 372–379. 25644361 10.3945/jn.114.195438

[42] Food and Agriculture Organization (2013) *Dietary Protein Quality Evaluation in Human Nutrition. Report of an FAO Expert Consultation*. Rome: FAO.

[43] Van Rossum CTM, Fransen HP, Verkaik-Klooseterman J, (2011) Dutch National Food Consumption Survey 2007–2010: Diet of Children and Adults Aged 7 to 69 Years. Bilthoven: RIVM.

[44] Westhoek H, Lesschen JP, Leip A, (2016) Nitrogen on the Table: The Influence of Food Choices on Nitrogen Emissions and the European Environment. Edinburgh: Centre for Ecology and Hydrology (CEH).

[45] Hetherington M, Havermans RC, Blundell JE, (2013) Sensory-specific satiation and satiety In Satiation, Satiety and the Control of Food Intake, pp. 253–269 [JE Blundell and F Bellisle, editors] Cambridge: Woodhead Publishing.

[46] Dias J, Huelvan C, Dinis MT, (1998) Influence of dietary bulk agents (silica, cellulose and a natural zeolite) on protein digestibility, growth, feed intake and feed transit time in European seabass (*Dicentrarchus labrax*) juveniles. Aquat Living Resour 11, 219–226.

[47] de Beukelaar MFA, Zeinstra GG, Mes JJ, (2019) Duckweed as human food. The influence of meal context and information on duckweed acceptability of Dutch consumers. Food Qual Prefer 71, 76–86.

